# *Aedes aegypti* Mosquitoes Exhibit Decreased Repellency by DEET following Previous Exposure

**DOI:** 10.1371/journal.pone.0054438

**Published:** 2013-02-20

**Authors:** Nina M. Stanczyk, John F. Y. Brookfield, Linda M. Field, James G. Logan

**Affiliations:** 1 Biological Chemistry and Crop Protection Department, Rothamsted Research, Harpenden, Hertfordshire, United Kingdom; 2 Centre for Genetics and Genomics, School of Biology, University of Nottingham, Nottingham, United Kingdom; 3 Department of Disease Control, London School of Hygiene and Tropical Medicine, London, United Kingdom; University of Crete, Greece

## Abstract

DEET (*N,N*-Diethyl-*m*-toluamide) is one of the most widely used mosquito repellents. Although DEET has been shown to be extremely effective, recent studies have revealed that certain individual insects are unaffected by its presence. A genetic basis for this has been shown in *Aedes aegypti* mosquitoes and the fruit fly *Drosophila melanogaster*, but, for the triatomine bug, *Rhodnius prolixus*, a decrease in response to DEET occurred shortly after previous exposure, indicating that non-genetic factors may also be involved in DEET “insensitivity”. In this study, we examined host-seeking behaviour and electrophysiological responses of *A. aegypti* after pre-exposure to DEET. We found that three hours after pre-exposure the mosquitoes showed behavioural insensitivity, and electroantennography revealed this correlated with the olfactory receptor neurons responding less to DEET. The change in behaviour as a result of pre-exposure to DEET has implications for the use of repellents and the ability of mosquitoes to overcome them.

## Introduction

The insect repellent *N,N*-diethyl-*m*-toluamide (DEET) is one of the most commonly used repellents worldwide [1]. However, despite its common use over the last 60 years, and evidence that it can repel 100% of mosquitoes in the laboratory, semi-field and field tests [2]–[4], there are several studies suggesting that certain individual insects are not repelled by DEET. For example, a small proportion of individuals in populations of *Aedes aegypti* mosquitoes and *Drosophila melanogaster* fruit flies will move towards an attractant despite the presence of DEET, a genetic “insensitivity” which can be selected for in the population [5]–[8], and which corresponds to changes in the function of the peripheral olfactory system [8]. However, in a recent study, the triatomine bug, *Rhodnius prolixus*, showed a decrease in behavioural repellency after continuous stimulation with DEET [9], indicating that other, non-genetic, factors may play a role in preventing insects from responding to DEET.

It has been shown that changes in behavioural responses by insects to compounds can occur through forms of ‘conditioning’ or ‘learned behaviours’ that are not genetically determined. Conditioning has been shown in *R. prolixus* [10], [11], *D. melanogaster* [12], and the parasitic wasp *Microplitis croceipes* [13]. For mosquitoes, some studies have found no evidence for behavioral adaptation [14], [15], while more recent work has demonstrated conditioning to odours [16]–[18]. Other investigations have shown that mosquitoes have a preference for returning to hosts they have successfully fed on previously [19]. These preferences are not passed on to their offspring and are therefore likely to be due to learned behaviour [20]. It has also been shown that mosquitoes return to sites where they have previously oviposited [21], [22], demonstrating that they can adapt their behaviour based on previously successful events. Interestingly, mosquitoes which emerge from eggs in sites where repellents are present have been shown to return to the same oviposition site, unaffected by the presence of the repellent [23], [24], suggesting that they can overcome repellents when accustomed to them or when they are associated with a reward.

In some studies on insects which change their behaviour after exposure to a compound, the responses of the olfactory receptor neurons (ORNs) on the antennae were altered [25]–[27], suggesting that pre-exposure to certain olfactory stimuli can modulate the peripheral olfactory system. Since DEET has been shown to be detected by ORNs on the antennae of mosquitoes [8], [28]–[30], it is possible that pre-exposure to this compound could alter behavioural responses through this mechanism.

Repeated exposure of mosquitoes to a repellent is likely to occur in situations where more than one host may be treated with a repellent, and mosquitoes feed multiple times during their lifespan. If pre-exposure to DEET does negatively affect the behaviour of mosquitoes, this would have major implications for how repellents should be evaluated and used for optimum personal protection. To our knowledge this has never been examined for mosquitoes.

In order to investigate the effect of pre-exposure to DEET on *A. aegypti*, we repeatedly exposed females to DEET and determined their subsequent behavioural and/or the electrophysiological responses.

## Results

Four separate experiments were performed to determine whether pre-exposure to DEET affected the behavioural and/or olfactory responses of mosquitoes to DEET when applied to a human arm, or when applied to an artificial heat source (to remove the effects of human volatiles). A summary of the experiments is given in Table 1.

**Table 1:** Description of experiments and the treatments tested with female *Aedes aegypti* mosquitoes.

	Treatment at 0h	Treatment at 3h	Tested with EAG
*Experiment 1*	−	CA	Random (s+i)
	CA	CA	−
	CA	DA	i
	−	DA	i
	DA	DA	s,i
*Experiment 2*	−	H	−
	H	H	−
	H	HD	−
	−	HD	−
	HD	HD	−
*Experiment 3*	−	CA	−
	−	DA	−
	DA	DA	−
	D	DA	−

Twenty mosquitoes were tested with a treatment (0 hr) and then re-tested after 3 hours. Experiment 1 tested against a control arm (0.5 ml ethanol) (CA) or DEET on an arm (0.5 ml, 20%) (DA). N = 10. Individuals collected for EAG were sensitive (s) or insensitive (i) to DEET, or collected at random. Experiment 2 tested a nylon control on a heat source (0.5 ml redistilled hexane) (H), or DEET on a section of nylon on a heat source (0.5 ml, 20% in redistilled hexane) (HD). N = 10. Experiment 3 tested DEET on a section of nylon tights with no arm present (0.5 ml, 20% DEET) (D). N = 10.

### Do mosquitoes change their behaviour after pre-exposure to DEET on a human arm?

**Figure 1:**
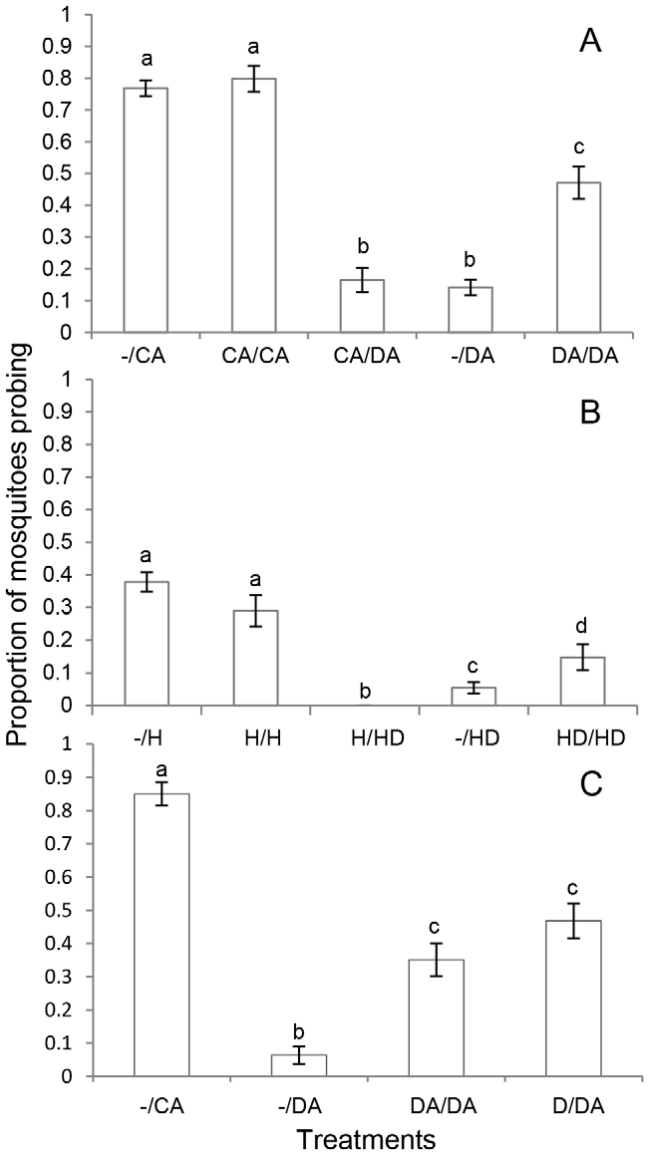
Behavioural repellency on second exposure. Proportion of female *Aedes aegypti* mosquitoes probing in response to a treatment on first exposure (treatment 1) or to a second treatment after pre-exposure to a treatment 3 hours previously (treatment 1/treatment 2). Treatments were **A**: a control arm (CA) (0.5 ml ethanol), a DEET treated arm (DA) (0.5 ml, 20% in ethanol); **B**: a hemotek heating device with nylon control (H) (0.5 ml hexane), a hemotek device with nylon spotted with DEET (HD) (0.5 ml, 20% in redistilled hexane); **C**: a section of nylon spotted with DEET with no other stimulus (D). Means are ± SEM. Means with different letters are significantly different from each other (p<0.05).

We examined whether female *A. aegypti* mosquitoes would change their behaviour when tested twice with a DEET treatment on a human arm. Mosquito responses were determined using an arm-on-cage repellency assay [8], during which mosquitoes which attempted to probe despite the presence of DEET were considered insensitive. Mosquitoes probing in response to DEET on an arm when first exposed were removed from the experiment, thus, mosquitoes probing on second exposure to DEET were all initially sensitive to DEET and had altered their behaviour. We found that previously DEET-sensitive females, which were exposed again to an arm treated with DEET (DA/DA), landed and probed significantly more on the second DEET exposure than mosquitoes tested for the first time with DEET (−/DA), or tested with DEET following exposure to a control arm (CA/DA) (p<0.001) (Fig. 1A). However, the proportion of mosquitoes probing on second exposure to DEET was still lower than the response to the untreated arm (−/CA) (p<0.001). Mosquitoes did not change their behaviour to the untreated control arm if pre-exposed to it (CA/CA). There was also no significant difference between the number of mosquitoes probing in response to DEET upon first exposure (−/DA) and to DEET tested 3 h after pre-exposure to the untreated control arm (CA/DA).

### Do mosquitoes change their behaviour after pre-exposure to DEET without the presence of an arm?

To eliminate the possibility of an interaction between host volatiles and DEET being involved in the observed changes in behavioural responses, we tested the mosquitoes with an artificial heating device in place of the arm (Fig. 1B). In this experiment, mosquitoes also probed significantly more in response to DEET upon second exposure (HD/HD) than when exposed to DEET for the first time (−/HD) (p = 0.016), although the proportion probing was still lower than the responses to the artificial heat source control (−/H) (p<0.001). There was no significant difference in response between mosquitoes at first and second exposure to the artificial heat source control (H/H). However, mosquitoes exposed to DEET on the heating device 3 h after pre-exposure to the artificial heat source control (H/HD) probed significantly less than in response to all other treatments (p = 0.001).

### Do mosquitoes change their behaviour after pre-exposure to DEET without the presence of an attractant?

When initially exposed to DEET without the presence of a human arm or a heat source, mosquitoes showed an increased attraction to a human arm with DEET on it (D/DA) compared with mosquitoes presented with this treatment for the first time (−/DA) (p<0.001) (Fig. 1C). This increased level of attraction on second exposure was not significantly different from the response of mosquitoes pre-exposed to DEET on an arm (DA/DA).

### Can altered behavioural responses to DEET be explained by changes in antennal olfactory responses?

**Figure 2:**
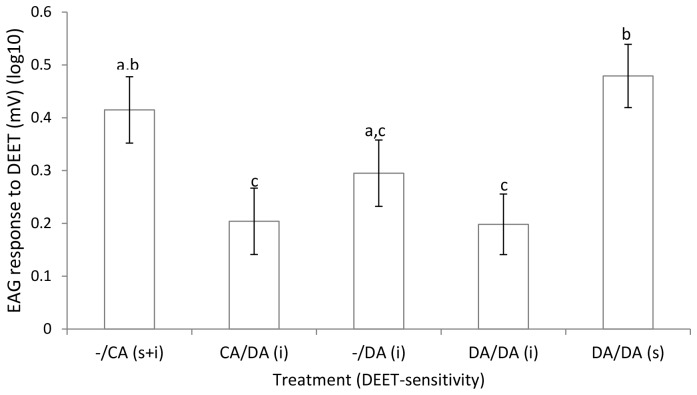
Olfactory responses of DEET-sensitive and insensitive mosquitoes. EAG responses of female *Aedes aegypti* showing behavioural DEET-sensitivity (s) or DEET-insensitivity (i) in experiment 1. Mosquitoes of unknown DEET-sensitivity (s+i) were collected from cages tested with a control arm. DEET-insensitive females were collected from cages tested with DEET on an arm (DA), tested first with a control arm and then DEET on an arm (CA/DA), and tested with DEET on an arm then retested with DEET on an arm (DA/DA). DEET-sensitive mosquitoes were also collected from cages tested with DEET on an arm and then retested with DEET on an arm (DA/DA). Means are ± SEM. Means with different letters are significantly different from each other (p<0.05).

To determine whether the antennal olfactory system was involved in the altered behavioural responses to DEET, we looked at EAG responses following the behavioural tests. This showed that there were no significant differences in EAG responses to DEET between any of the DEET-insensitive mosquitoes tested from the groups which had been treated at 3 hrs with DEET after pre-exposure to either a control arm (CA/DA*i*) or an arm with DEET on it (DA/DA*i*), or those with only an initial exposure to *DEET* (−/DA*i*) (Fig. 2). However, the DEET-sensitive mosquitoes, collected after a second exposure to DEET (DA/DA*s*), had a significantly greater response to DEET than the three groups of DEET-insensitive mosquitoes (p = 0.001, p = 0.019, p<0.001 respectively). The response to DEET of the control group (−/CA) was not significantly different from the DEET-sensitive mosquitoes or the DEET-insensitive mosquitoes collected during initial exposure to DEET (−/DA*i+s)*, but was significantly greater than the response of the DEET-insensitive mosquitoes exposed to DEET after a control arm (CA/DA*i*) (p = 0.01) or a DEET arm (DA/DA*i*) (p = 0.006).

## Discussion

The genetic insensitivity to DEET found in previous studies [5]–[8] cannot be the cause of the change in behaviour of *A. aegypti* which occurred over a short, three hour, period in the experiments reported here. Our observed increase in insensitivity to DEET on a second exposure, by previously DEET-sensitive mosquitoes, initially suggested they may have adapted to DEET, possibly by associating it with the presence of a host arm, and were able to ‘overcome’ the natural repellent effect. This would be consistent with other studies showing that mosquitoes can learn to respond differently to odours to maximise feeding success [19], [20]. Both *Culex quinquefasciatus* mosquitoes [17], the parasitic wasp *M. croceipes* [13], and the triatomine bug *R. Prolixus* [31]–[33] can learn to associate a neutral odour with a food source through Pavlovian conditioning, and adapt their host-seeking preferences accordingly. In *C. quinquefasciatus* this conditioning could last for up to 24 hours in colony mosquitoes, though fewer mosquitoes responded over time [18]. However, in our study, altered behaviour towards DEET did not result in a reward (i.e. the mosquitoes were not given a blood meal) other than the ability to move towards a human arm/heat source, and this behaviour occurred even when there was no host-related stimulus present. Interestingly, mosquitoes showed increased repellency by DEET on the artificial heat source when pre-exposed to the heat, which was not seen towards DEET on an arm after pre-exposure to the control arm (Fig. 1A, B). The presence of human volatiles with the DEET stimulus may have been a greater incentive for the mosquitoes to persist in host seeking when re-exposed, compared to the weaker attraction of heat alone. Overall, the increased response to a second treatment with DEET on an attractive stimulus, after pre-exposure to DEET with no attractant present, indicates that the learned behaviour is not by association with an attractant as was found in other studies with host-seeking insects [11], [18], [33]. It is instead a direct response to a single exposure to the DEET, Such habituation to DEET has been shown in *R. prolixus*, where continuous stimulation led to 10–20 minutes of reduced repellency [9]. Thus it seems likely that in our experiments increased DEET-insensitivity results from sensory adaptation or habituation, whereby there is an decrease in response to a stimulus (in this case, DEET) after repeated exposure [34], [35].

The phenomenon of insects changing their response to a compound after pre-exposure or conditioning has been investigated with EAG in *D. melanogaster, M. croceipes, Apis mellifera and Protophormia terraenovae* [25]–[27], [36], [37]. In some cases no changes in EAG were found, even though the insects were exhibiting changed behavioural responses [36], [37]. In *D. melanogaster*, the behavioural change was suggested to be caused by a reduction in the volume of glomeruli, and corresponding synapse loss, over a week's exposure to the chemical. In contrast, other trials with *D. melanogaster, A. mellifera* and *P. terraenovae*, all using pre-exposures of less than 60 seconds, a decrease in EAG responses to the compounds was observed [25]–[27]. For *D. melanogaster*, the insects were no longer behaviourally repelled by a repellent, and the EAG decrease only lasted for a brief time, with responses returning to half the normal level in four minutes [25]. In our study, the mosquitoes that had become behaviourally insensitive to DEET also showed a lower EAG response to the repellent (Fig. 2), in contrast to the *D. melanogaster* study where no change in EAG responses was seen [36]. This supports the possibility that habituation is occurring in our study, as exposure to a chemical for a week, as in the *D. melanogaster* study, would give time for different changes causing behavioural alteration to occur, such as the loss of synapses, compared to the changes induced in the peripheral olfactory system after brief exposure [25]–[27].

In work on *P. terraenovae*, the authors concluded that non-associative learning processes, such as habituation, occurred with repeated doses of a repellent [27]. When re-tested with the repellent, approximately 50% of flies no longer responded, which is similar to the level of behavioural DEET-insensitivity found in our study on a second exposure. This might suggest that the same mechanism may be responsible for the behavioural changes. However, in *D. melanogaster, P. terraenovae*, and indeed in vertebrates, habituation causing a change in response only lasts for a few minutes to half an hour [25], [27], [38], after which the responses return to normal (dishabituation, which is a key characteristic of habituation) [35], [39]. This is in contrast to the altered behavioural and EAG responses to DEET seen in our study with *A. aegypti*, where the effect lasted for at least 3 hours. It is possible that sensory adaptation and habituation vary between species, and last longer in *A. aegypti* than in *R. prolixus, D. melanogaster or P. terraenovae*, and experiments carried out over longer time periods would ascertain if *A. aegypti* responses did return to normal. However, there is evidence in the nematode *Caenorhabditis elegans* for two separate causes of decreased response to an odour, with low concentrations resulting in habituation, where the responses return to normal, and high concentrations resulting in sensory adaptation, with responses not returning to normal [39]. In the nematodes the cause of the adaptation was thought to be sensory or receptor fatigue. The same could be true for the mosquitoes in our study, if the ORs are desensitized to DEET after first exposure. This would, however, have to occur differentially between the mosquitoes which altered after first exposure to show behavioural insensitivity to DEET, and those which did not.

If, as shown here, mosquitoes can change their response to a repellent after pre-exposure, then caution should be taken when testing insects multiple times in behavioural repellency bioassays. Methods which retest the same mosquitoes are commonly used, and could be affected by the adaptive behaviour shown in our study [4], [40]–[43]. It should also be determined whether the adaptive behaviour occurs in an arm-in-cage experiment to find out if mosquitoes should not be re-used in repellency tests. DEET has been shown to be 100% effective for up to 5 hours in arm-in cage tests [3], possibly due to the higher concentrations used (28%) or different behaviour triggered on more frequent, every 10–15 min, exposure. When tested at similar concentrations to those in our study, complete repellency lasted for under 4 hours, and insensitivity to the repellent could have occurred after this time. The time for the receptors to return to normal should also be investigated to determine whether dishabituation occurs, or if the change in olfaction is due to receptor fatigue or indeed any other cause. It would be interesting to discover other compounds which may have this effect on mosquito olfaction, and if it could be artificially induced to lower responses to attractants. Investigating the ORNs involved and finding the mechanism responsible may lead to improved control methods. Perhaps the most urgent need is to examine whether the insensitivity also occurs in ‘semi-field’ or ‘field’ situations to determine whether mosquitoes might be less sensitive to repellents if they encounter them for the second time. As research in the field suggests repellents are an important part of transmission prevention strategies in communities [44]–[45], and that some insecticides have repellent properties [46], the likelihood of a mosquito encountering a repellent multiple times is increased. *A. aegypti*, while primarily dawn and dusk feeders, will continue to feed throughout the day, particularly in shaded or forested areas. Thus the behavioural insensitivity seen here after 3 hours is relevant to their host-seeking period. The effect of pre-exposure, and the relevance to the cycle of feeding activity, may differ for different mosquitoes depending how long the insensitivity lasts. In the field, the concentrations of DEET applied as personal protection would also diminish over time, which could increase the proportion of mosquitoes altering their behaviour. After three hours DEET is still 100% effective, but over longer time periods decreasing effectiveness might have a greater impact. It is therefore important to study this phenomenon over longer times, as this would have clear implications for use of repellents for personal protection, and the use of repellents has been shown to have a direct impact on disease transmission [47].

## Materials and Methods

### Insects

The mosquitoes used in this study were *A. aegypti* (refm strain obtained from the Liverpool School of Tropical Medicine) reared in 30×30×30 cm Bugdorm 1 cages (Megaview®) in rooms maintained at 27.5°C±1°C, 60–80% relative humidity, and a 12∶12 light:dark cycle. Larvae were reared on Tetramin® tropical fish flakes, and adults were fed on 10% sucrose solution. Females were fed with sheeps' blood using a Hemotek® system. The females used in behavioural experiments were 5–12 days old and had not been blood-fed. For the electrophysiological experiments, females tested were selected in experiment 1 for insensitivity or sensitivity to DEET.

### Experiment 1 Do mosquitoes change their behaviour after pre-exposure to DEET on a human arm?

Female *A. aegypti* were tested for their response to DEET (97%, Aldrich) using an arm-on-cage repellency assay [8]. The treated arm was held 1.5 cm above a cage of 10 mosquitoes, separated by a section of metal mesh, and the behaviour of the mosquitoes observed for 2 minutes. Mosquitoes which attempted to probe the mesh beneath the arm when DEET was present were considered insensitive. An initial test with either 0.5 ml ethanol (control) or 0.5 ml DEET (20%) on the arm (rubbed onto the arm between wrist and elbow on a surface area of 506 cm, and allowed 30 s to evaporate) was carried out at 0 h, and then 3 h later the same 10 mosquitoes were retested with either ethanol or DEET on the arm. At 3 h, cages of mosquitoes which had been prepared, but not tested, at 0 h were tested with an ethanol or DEET arm to control for variability in response over time. There were ten replicates for each treatment, with each treatment tested once within a block. Test cages were randomly placed each time in a controlled environment to ensure no bias. Mosquitoes which were tested with DEET on an arm at 0 h and were insensitive were removed from the cage by mouth aspirator (with minimal disturbance to other mosquitoes), so that only previously sensitive mosquitoes were retested with DEET at 3 h to see if their response had changed. Thus, the number of mosquitoes probing when retested with DEET changed their sensitivity (i.e. changed from DEET-sensitive to insensitive). At the end of each of the 3 h trials, mosquitoes were removed (by mouth aspirator) from the cage to be used in experiment 4.

### Experiment 2 Do mosquitoes change their behaviour after pre-exposure to DEET without the presence of human volatiles?

To eliminate the effect of human volatiles during either pre-exposure or re-testing with DEET, an experiment was carried out replacing the arm with heat from a Hemotek® artificial heating system with a section of nylon (4 cm unstretched, Boots brand 97% nylon, 3% LYCRA®, small/medium, nude, Denier 10 tights) covering the heating block reservoir (3.5 cm diameter, stainless steel). The nylon was treated with either redistilled hexane as a control (0.5 ml), or with DEET (0.5 ml, 20% in redistilled hexane), spotted evenly over the material and allowed 2 min to evaporate before being stretched over the Hemotek® and held in place with an ‘o’ ring. The Hemotek® reservoir was maintained at 27°C, and positioned 0.5 cm above the mesh of the experimental cage. Methods were as in experiment 1, with cages tested initially with a control or DEET treatment on the Hemotek®, and retested 3 h later with a control or DEET treatment, with the inclusion of new, unexposed controls. For the duration of this treatment, no volunteer was present in the room to avoid contact with human volatiles, and responses were recorded by video camera. Mosquitoes which were insensitive to DEET initially were therefore unable to be removed from the cage, and were retested at 3 hrs.

### Experiment 3 Do mosquitoes change their behaviour after pre-exposure to DEET without the presence of a heat stimulus?

To eliminate any effect of pre-exposure to the heat from either the human arm or the Hemotek® artificial heating system an experiment was carried out with initial exposure to a section of nylon spotted evenly with DEET (as above). The nylon was placed over the mesh on the cage at 0 h and left for a 2 min exposure of the mosquitoes before being removed. For the duration of this treatment, no volunteer was present in the room, so that no human volatiles or heat source were presented to the cage. No mosquitoes were visibly insensitive to DEET during this treatment, as there was no attractant present. At 3 h the cage was tested with DEET on an arm.

### Experiment 4 Can altered behavioural responses to DEET be explained by changes in antennal olfactory responses?

Antennae for electroantennography (EAG) were prepared as described by Logan *et al.* [48]. Signals were recorded and analyzed (amplified ×10,000) using a software package (EAG v2.6, Syntech®, The Netherlands). The test compound (10 μL in distilled hexane) was applied to a strip of filter paper, and 30 s was allowed for the solvent to evaporate. The filter paper was then placed in a glass pipette cartridge and, using a stimulus controller, a 2 s air puff was passed into the continuous airstream through a hole in the glass tube at a 7 cm distance and the response to the stimulus was recorded. Fresh preparations were used for each recording.

Each mosquito was tested with three treatments: a control (hexane), a ‘standard’ compound known to elicit an electrophysiological response (methyl salicylate 1×10^−4^ g), and DEET 1×10^−3^ g. The control and standard stimuli were applied at the beginning of each test and again after DEET had been tested, to determine the mosquito's ability to respond and to establish baseline responses. If the mosquito showed no response to methyl salicylate it was classified as a non-responder and not tested with DEET. Two minutes were left between each recording.

Mosquitoes were collected at the end of experiment 1, classified as either behaviourally sensitive or insensitive to DEET, or collected at random from control cages. All mosquitoes were tested with EAG within 3 h following the behavioural experiment.

### Statistics

For the behavioural experiments, the number of mosquitoes successfully probing during each treatment was analysed using regression analysis in a generalised linear model (GLM) in Genstat® (12th edition), modelling binomial proportions with a logit transformation blocking by replicate and day. This was used to obtain predicted means and standard errors of the means (SEMs). Differences were deemed to be significant when the difference between means was greater than the least significant difference (LSD).

EAG responses were corrected by dividing the response in millivolts by the average of the control values before and after the stimulation of each test treatment. Thus, the control value was 1 and the response was expressed as a proportion of 1. The mean responses between treatments were compared by using a one-way ANOVA in Genstat® (12th edition), using replicates as blocks. The data were log (base10) transformed. Differences were deemed to be significant when the difference between means was greater than the LSD.
